# 

**Published:** 1906-12-29

**Authors:** 


### BOOKS RECEIVED.

T. FISHER UNWIN.

"How to Keep Well." By C. Stanford Read, M.B.

SPOTTISWOODE AND CO., LTD.

"Mountain Sickness and its Probable Causes." By T. G. Longstaff, M.A., D.M.

C. GRIFFIN AND CO., LTD.

"Official Year-book of the Scientific and Learned Societies, 1906."

J. B. LIPPINCOTT CO.

"Post-mortem Pathology." By H. W. Cattell, M.D. 3rd edition.

THE SCIENTIFIC PRESS, LTD.

"The Nurses' Enquire Within."

**Notice to Advertisers and Subscribers.**

All Advertisements, Orders for Copies of The Hospital, and Business Communications should be addressed to **THE MANAGER (Not to the Editor),** The Hospital, 28 & 29 Southampton Street, Strand London, W.C.

The Cost of Subscription, post free, is as follows:—Per week, 3½d.; per quarter, 3s. 3d.; per half-year, 6s. 6d.; per annum, 13s. Foreign and Colonial:—Per annum, 19s. 6d., payable, in all cases, in advance.

By post, 1/1

**HOW TO BECOME A CERTIFIED MIDWIFE.**
By E. L. C. Appel, M.B., B.S., B.Sc.Lond.

**SIMPLE INTRODUCTORY LESSONS IN MIDWIFERY.**
By M. Loane.

**THE NURSING OF SICK CHILDREN.**
By Dr. Burnet.

**THE MODERN NURSING OF CONSUMPTION.**
By Dr. Jane H. Walker.

**SIMPLE SANITATION:**
**The Practical Application of the Laws of Health to Small Dwellings.**
By M. Loane. With an Introductory Chapter on Sanitary Legislation by Dr. A. Mearns Fraser, Medical Officer of Health, Portsmouth.

**PRACTICAL HINTS ON DISTRICT NURSING.**
By Miss Amy Hughes.

**FEVERS AND INFECTIOUS DISEASES:**
**Their Nursing and Practical Management.**
By William Harding, M.D.Ed., M.R.C.P. Lond., Author of "Mental Nursing," &c.

**NOTES ON PHARMACY AND DISPENSING FOR NURSES.**
New and Revised Edition.
By C. J. S. Thompson.

### 1/6 NURSING HANDBOOKS

By Post, 1/8

**SURGICAL INSTRUMENTS AND APPLIANCES USED IN OPERATIONS.**
**An Illustrated and Classified List, with Explanatory Notes.**
By Harold Burrows, M.B.Lond., B.S., F.R.C.S.
New Edition, Revised and Enlarged.

**ART OF FEEDING THE INVALID.**
By a Medical Practitioner and a Lady Professor of Cookery.
New and Popular Edition, with Special Chapter on Diet for Consumptives.

**THE CARE OF CHILDREN.**
**Practical Hints for Mothers and Nurses at Home and Abroad.**
By Robt. J. Blackham, D.P.H.Lond., C.B.
Revised and Enlarged Edition.

**LECTURES ON HOME NURSING FOR THE POOR.**
By a District Nurse.
Illustrated.

**OUTLINES OF ROUTINE IN DISTRICT NURSING.**
By M. Loane, Superintendent of District Nurses, Portsmouth.
1/9 post free.

**OUTLINES OF INSANITY.**
**A Popular Treatise on the Salient Features of Insanity.**
By Francis H. Walmsley, M.D.
Popular Edition.

**NURSING OF TROPICAL FEVERS.**
By S. F. Pollard, late Sister, Army Nursing Reserve.
The Work is based upon the Personal Experience of the Author Abroad.

### THE SCIENTIFIC PRESS, Ltd.,

**Publishers & Booksellers, 28 & 29 SOUTHAMPTON ST., STRAND, LONDON, W.C.**

*COMPLETE CATALOGUE POST FREE ON APPLICATION.*

